# Development of Risk Prediction Models for Severe Periodontitis in a Thai Population: Statistical and Machine Learning Approaches

**DOI:** 10.2196/48351

**Published:** 2023-12-14

**Authors:** Htun Teza, Anuchate Pattanateepapon, Attawood Lertpimonchai, Prin Vathesatogkit, Gareth J McKay, John Attia, Ammarin Thakkinstian

**Affiliations:** 1 Department of Clinical Epidemiology and Biostatistics Faculty of Medicine Ramathibodi Hospital Mahidol University Bangkok Thailand; 2 Department of Periodontology Faculty of Dentistry Chulalongkorn University Bangkok Thailand; 3 Center of Excellence in Periodontal Disease and Dental Implant Chulalongkorn University Bangkok Thailand; 4 Department of Medicine Faculty of Medicine Ramathibodi Hospital Mahidol University Bangkok Thailand; 5 Centre for Public Health, School of Medicine Dentistry and Biomedical Sciences Queen’s University Belfast Belfast United Kingdom; 6 School of Medicine and Public Health Hunter Medical Research Institute University of Newcastle, New Lambton NSW Australia

**Keywords:** periodontitis, prediction, machine learning, repeated measures, panel data

## Abstract

**Background:**

Severe periodontitis affects 26% of Thai adults and 11.2% of adults globally and is characterized by the loss of alveolar bone height. Full-mouth examination by periodontal probing is the gold standard for diagnosis but is time- and resource-intensive. A screening model to identify those at high risk of severe periodontitis would offer a targeted approach and aid in reducing the workload for dentists. While statistical modelling by a logistic regression is commonly applied, optimal performance depends on feature selections and engineering. Machine learning has been recently gaining favor given its potential discriminatory power and ability to deal with multiway interactions without the requirements of linear assumptions.

**Objective:**

We aim to compare the performance of screening models developed using statistical and machine learning approaches for the risk prediction of severe periodontitis.

**Methods:**

This study used data from the prospective Electricity Generating Authority of Thailand cohort. Dental examinations were performed for the 2008 and 2013 surveys. Oral examinations (ie, number of teeth and oral hygiene index and plaque scores), periodontal pocket depth, and gingival recession were performed by dentists. The outcome of interest was severe periodontitis diagnosed by the Centre for Disease Control–American Academy of Periodontology, defined as 2 or more interproximal sites with a clinical attachment level ≥6 mm (on different teeth) and 1 or more interproximal sites with a periodontal pocket depth ≥5 mm. Risk prediction models were developed using mixed-effects logistic regression (MELR), recurrent neural network, mixed-effects support vector machine, and mixed-effects decision tree models. A total of 21 features were considered as predictive features, including 4 demographic characteristics, 2 physical examinations, 4 underlying diseases, 1 medication, 2 risk behaviors, 2 oral features, and 6 laboratory features.

**Results:**

A total of 3883 observations from 2086 participants were split into development (n=3112, 80.1%) and validation (n=771, 19.9%) sets with prevalences of periodontitis of 34.4% (n=1070) and 34.1% (n=263), respectively. The final MELR model contained 6 features (gender, education, smoking, diabetes mellitus, number of teeth, and plaque score) with an area under the curve (AUC) of 0.983 (95% CI 0.977-0.989) and positive likelihood ratio (LR+) of 11.9 (95% CI 8.8-16.3). Machine learning yielded lower performance than the MELR model, with AUC (95% CI) and LR+ (95% CI) values of 0.712 (0.669-0.754) and 2.1 (1.8-2.6), respectively, for the recurrent neural network model; 0.698 (0.681-0.734) and 2.1 (1.7-2.6), respectively, for the mixed-effects support vector machine model; and 0.662 (0.621-0.702) and 2.4 (1.9-3.0), respectively, for the mixed-effects decision tree model.

**Conclusions:**

The MELR model might be more useful than machine learning for large-scale screening to identify those at high risk of severe periodontitis for periodontal evaluation. External validation using data from other centers is required to evaluate the generalizability of the model.

## Introduction

Periodontitis, one of the most common oral diseases, is a major cause of tooth loss in adult life [1] with a prevalence of 11.2% globally and 15% to 20% in Asia [2]. It is a complex inflammatory disease affecting supportive structures around the tooth, resulting in loosening and eventual loss [3]. This leads to decreased dental occlusion, digestive ability, and quality of life. In addition to oral manifestations, it is also associated with other inflammatory or systemic diseases [4], including atherosclerotic vascular disease [5], diabetes mellitus, chronic kidney disease [6], chronic obstructive pulmonary disease, rheumatoid arthritis, Alzheimer disease, and erectile dysfunction [7].

Severe periodontitis is characterized by the loss of alveolar bone height, which is asymptomatic until the tooth becomes mobile. Radiographs are usually used as the standard tool for diagnosis along with a full mouth examination by dentists, both of which are time- and resource-intensive, especially in public health sectors constrained by the large number of participants that require examination. The impact on resource allocation can, in part, be addressed through the use of screening tools, such as risk prediction models, to identify those at high risk of severe periodontitis.

Identification of severe periodontitis risk factors has been achieved largely through cross-sectional investigations that have evaluated demographic features, risk behaviors, and oral characteristics [8-13]. Inclusion of both demographic and oral features as predictors has been reported to outperform models composed of either feature alone [9]. Furthermore, the addition of saliva biomarkers to established risk factors further improved performance [11], but including such parameters necessitates the requirement for oral examination, which is contradictory to the purpose of a screening tool (for reducing time and resources). However, the majority of studies have used cross-sectional data, which fail to capture the complex relationship between the features and outcomes in contrast to longitudinal investigations that reflect both interindividual and intraindividual dynamics [14].

Machine learning approaches for disease risk prediction have been proposed, which might perform better in dealing with multidimensional interactions, collinearity between features, and nonlinear relationships than the traditional statistical models [15-17]. Several machine learning algorithms, such as support vector machines, decision trees, and artificial neural networks, have also been reported to improve the diagnosis of periodontal disease [18,19]. The performance of artificial neural networks was also considered [20] with the inclusion of probing pocket depth (PPD) as a predictor. However, PPD requires a comprehensive periodontal examination, which is time- and resource-intensive. Applying machine learning screening models without PPD may help reduce the number of patients requiring dental examination and associated resource commitments. As such, the aim of this study was to use longitudinal data to compare the performance of statistical and machine learning approaches for periodontitis risk prediction.

## Methods

### Setting and Study Population

This study used data from the prospective Electricity Generating Authority of Thailand (EGAT) cohort study [21]. Dental examinations were performed in the 2008 and 2013 surveys.

### Participant Eligibility

Participants were included if they had received periodontal examinations in both surveys (2008 and 2013) regardless of having periodontitis at the baseline survey. Some participants were excluded if they did not receive periodontal examinations due to a refusal to participate, had systemic conditions that required antibiotic prophylaxis before dental examination (eg, congenital heart disease or valvular heart disease, a previous history of bacterial endocarditis or rheumatic fever, total joint replacement, and end-stage renal disease), or were fully edentulous.

### Clinical Features

#### Demographic and Clinical Characteristics

General demographic data (ie, age, gender, educational level, and income), behavioral data (ie, smoking status and alcohol consumption), underlying diseases (eg, diabetes mellitus, hypertension, chronic kidney disease), and lipid lowering medication information were collected by self-administered questionnaires at both time points. A physical examination including weight, height, and waist and hip circumference was also performed at the survey site. Laboratory tests included lymphocytes, uric acid, and lipid profiling.

#### Oral Features

Oral examinations included the number of teeth and oral hygiene index (plaque score) [22], which were carried out by the Department of Periodontology, Faculty of Dentistry, Chulalongkorn University. PPD and gingival recession (RE) were measured at 6 sites (buccal/labial, lingual/palatal, mesiobuccal, mesiolingual, distobuccal, and distolingual) on all fully erupted teeth except for third molars and retained roots. Centre for Disease Control–American Academy of Periodontology (CDC-AAP) criteria were used to classify severe periodontitis. PPD was defined as the distance from the coronal point of the gingival margin to the tip of a periodontal probe, and the RE was defined as the distance to the cementoenamel junction, with the clinical attachment level calculated by subtracting the RE from the PPD.

#### Outcome

The primary outcome of interest was severe periodontitis as defined by the CDC-AAP guidelines [23] at 2 or more interproximal sites with a clinical attachment level ≥6 mm (on different teeth) and 1 or more interproximal sites with a PPD ≥5 mm.

### Model Development

Among the included participants, the missing data rate was relatively low, ranging from 0.03% to 9.3% (Table S1 in Multimedia Appendix 1). Multiple imputation with chain equations (MICE) was applied to impute missing data assuming the data were missing at random (additional detail is provided in Multimedia Appendix 1). Given that repeatedly measured data were applied, a multilevel predictive mean matching method was applied for all continuous features using the *miceadds-3.13-12* R library. Features used to impute the missing data are presented in Table S2 in Multimedia Appendix 1. A total 5 imputations for MICE were constructed with 8 iterations each for estimation (Figure S1 in Multimedia Appendix 1). Distributions of features in complete-case and imputed data were almost the same (Table S3 and Figure S2 in Multimedia Appendix 1).

A total of 21 features, including demographic characteristics (age, gender, education level, and income), physical examinations (body mass index and waist to hip ratio), underlying diseases (diabetes mellitus, hypertension, dyslipidemia, and chronic kidney disease), risk behaviors (smoking status and alcohol drinking habits), oral features (number of teeth and plaque score), laboratory features (lymphocytes, uric acid, triglyceride, cholesterol, high density lipoprotein, and low density lipoprotein), and a lipid lowering drug, were considered as predictors. Of them, 9 features were included as categorical data with the rest as continuous data.

To the best of our knowledge, there are no explicit guidelines for sample size estimation for machine learning models, but previous studies have recommended that this be based on disease prevalence estimates [24]. According to the 8th National Oral Health Survey of Thailand (2017) [25], the adult prevalence of severe periodontitis in the Thai population was 26%. A total of 296 participants would therefore be required, assuming a type 1 error rate of 5% and a 95% CI, with 77 having severe periodontitis. Our data included 2086 participants that underwent periodontal examination, with 721 characterized as having severe symptoms, providing sufficient power.

We considered 4 models: a mixed-effects logistic regression (MELR), recurrent neural networks (RNN), a mixed-effects support vector machine (ME-SVM), and a mixed-effects decision tree (ME-DT). For mixed-effects approaches, a random intercept was fitted considering the effect of participants as random. The framework for model development is shown in Figure S3 in Multimedia Appendix 1. For the MELR [26], feature selection was performed based on the following steps suggested by Hosmer-Lemeshow [27]: (1) univariate analysis of MELR was performed and indicated that 15 out of the 21 features (ie, age, gender, education level, income, waist to hip ratio, diabetes mellitus, hypertension, smoking status, alcohol drinking habits, number of teeth, plaque score, lymphocytes, uric acid, triglyceride, and high density lipoprotein) had *P*≤.1 (Table S4 in Multimedia Appendix 1), which were then considered simultaneously in a multivariate MELR; (2) a stepwise process with forward selection was applied by including each of the 15 features into the MELR model one by one, and only significant features were kept in the final model; (3) 6 nonsignificant features (ie, body mass index, dyslipidemia, chronic kidney disease, cholesterol, low density lipoprotein, and lipid lowering drug) in the univariate analysis were also reconsidered to add in the final multivariate MELR model that contained only significant features, but none of them were significant, thus they were omitted; (4) interactions between significant features (eg, smoking and gender, smoking and plaque score, plaque score and diabetes) were considered but none were significant; and (5) odds ratios and 95% CIs of all significant features were estimated based on the final model.

A total of 21 features were considered in the machine learning models. For RNN [28], ME-SVM, and ME-DT [29], hyperparameter optimization was done using a random search of the hyperparameter sets followed by grid-search procedures. This process can be subject to unfocused random noise in data development and a failure to generalize. As such, a validation data set was used to assess model performance, and the hyperparameters were readjusted if overfitting was present. The RNN model was developed using Keras-2.4.3 [30] and TensorFlow-2.3.1 [31]. The final model specifications for RNN were 4 hidden layers with 62, 72, 72, and 62 simple RNN nodes with a Tanh activation function in feed-forward order with a dropout of 0.2 allocated between hidden and output layers. The output layer had 1 sigmoid node for binary classification. Binary cross entropy represented a loss function, with accuracy as a monitor metric. A learning rate of 0.01 and a batch size of 64 were applied for mini-batch optimization. A total of 10,000 epochs were used with early stopping due to time and resource constraints.

The ME-SVM included support vector regression developed within the *e1071-1.7.4* R library framework for fixed effects and a linear mixed model developed with *lme4-1.1.26* for random effects. Support vector regression here applies nu-regression [32], with a nu value of 0.5, a cost value of 0.1 as the penalty parameter for misclassifications, and a radial basis kernel function with a gamma value of 0.3. Similarly, ME-DT used the *rpart-4.1.16* library framework with a maximum tree depth of 18 and a minimum number of subjects for splitting of 20. Hyperparameter tuning was performed by leave-one-out (K-1) cross-validation.

The probability of having periodontitis was estimated by each model. Participants were classified as positive if the estimated probability was ≥0.35, as per the prevalence of periodontitis of our data. A contingency 2x2 table was then constructed comparing positive and negative classifications with actual periodontitis. The performance of each model was further evaluated by estimating sensitivity, specificity, accuracy, positive likelihood ratio (LR+), and *F*_1_-score. In addition, discrimination and calibration performances were also assessed using the area under receiver operating curves (AUC) and Brier scores. Values ranged from 0 to 1 for both, with a higher score being preferable for the AUC in contrast to a lower score for the Brier score.

All analyses were performed based on imputed data using STATA version 16.0 (StataCorp) for MELR, Python version 3.8.2 (Python Software Foundation) for RNN, and R version 4.02 (R Foundation for Statistical Computing) for ME-SVM and ME-DT.

### Ethical Considerations

This study was approved by the Human Research Ethics Committee, Faculty of Medicine Ramathibodi Hospital, Mahidol University (COA.MURA2020/1560). For the prospective EGAT cohort, all participation was voluntary and the participants gave written informed consent, including permission for secondary analyses of the collected data for necessary further studies. Identifications and personal information were encrypted and kept in databases that only the principal investigators could access.

## Results

A total of 2271 participants were initially included in the cohort in 2008, but only 2086 participants were followed up 5 years later in 2013. The key characteristics of the 2086 participants comparing those with and without periodontitis are reported in Tables 1 and 2. 71% (n=1482) of the participants were men. The mean age was 54.4 (SD 5.0) years, with the youngest being 43.7 years and the oldest being 70.3. 

Each participant was observed 1 to 2 times, and of the 3883 total observations included in this study, 47.2% (n=1834) of participants had a bachelor or higher degree, and 70.8% (n=2749) earned >50,000 baht (>US $1500) per month. Approximately 67.8% (n=2634) consumed alcohol and 16.7% (n=648) were current smokers at the time of observation. The prevalence of diabetes, hypertension, and dyslipidemia were 12.9% (n=499), 44.8% (n=1741), and 71.5% (n=2775), respectively. Data from both surveys were split into development (n=3112, 80.1%) and validation (n=771, 19.9%) sets [33] at the participant level to prevent data leakage (ie, participants were included in either the development or the validation set only). Participants in the development and validations sets had a prevalence of periodontitis of 34.4% (n=1070) and 34.1% (n=263), respectively (Table S6 in Multimedia Appendix 1).

**Table 1:** Gender of participants with and without observed severe periodontitis.

Demographic		All participants (n=2086)	Participants with severe periodontitis (n=721)	Participants with nonsevere periodontitis (n=1365)
**Gender, n (%)**				
	Men	1482 (71)	591 (82)	891 (65.3)
	Women	604 (29)	130 (18)	474 (34.7)

**Table 2:** Age, education level, income, and clinical characteristics of participants with and without observed severe periodontitis.

Characteristic		All observations (n=3883)	Observations of participants with severe periodontitis (n=1333)	Observations of participants with nonsevere periodontitis (n=2550)
Age (years), mean (SD)		54.4 (5.0)	55.0 (5.1)	54.0 (5.0)
**Education level, n (%)**				
	High school graduate or lower	767 (19.8)	406 (30.4)	361 (14.2)
	Vocational school graduate	1282 (33)	522 (39.2)	760 (29.8)
	Bachelor degree graduate	1519 (39.1)	341 (25.6)	1178 (46.2)
	Above bachelor degree	315 (8.1)	64 (4.8)	251 (9.8)
**Monthly income in Baht (US $), n (%)**				
	Less than 20,000 (US $600)	306 (7.9)	149 (11.2)	157 (6.2)
	Between 20,000-49,999 (US $600-$1499.97)	828 (21.3)	365 (27.4)	463 (18.1)
	More than 50,000 (US $1500)	2749 (70.8)	819 (61.4)	1930 (75.7)
Body mass index, mean (SD)		24.9 (3.7)	24.9 (3.7)	24.8 (3.7)
Waist to hip ratio, mean (SD)		0.9 (0.1)	0.9 (0.1)	0.9 (0.1)
Diabetes mellitus, n (%)		499 (12.9)	234 (17.5)	265 (10.4)
Hypertension, n (%)		1741 (44.8)	676 (50.7)	1065 (41.8)
Dyslipidemia, n (%)		2775 (71.5)	954 (71.6)	1821 (71.4)
Chronic kidney disease, n (%)		293 (7.5)	105 (7.9)	188 (7.4)
**Smoking status, n (%)**				
	Nonsmoker	2092 (53.9)	498 (37.3)	1594 (62.5)
	Exsmoker	1143 (29.4)	446 (33.5)	697 (27.3)
	Current smoker	648 (16.7)	389 (29.2)	259 (10.2)
**Alcohol consumption, n (%)**				
	Nonconsumer	1249 (32.2)	309 (23.2)	940 (36.8)
	Occasional consumer	695 (17.9)	242 (18.1)	453 (17.8)
	Frequent consumer	1939 (49.9)	782 (58.7)	1157 (45.4)
Number of present or remaining teeth, mean (SD)		23.4 (4.9)	22.1 (5.4)	24.1 (4.5)
Plaque score (%), mean (SD)		70.9 (21.5)	78.5 (18.8)	66.9 (21.7)
Lymphocytes (mm^3^), mean (SD)		2156.3 (623)	2224.5 (636.9)	2120.6 (612.7)
Uric acid (mg/dL), mean (SD)		5.9 (1.4)	6.1 (1.4)	5.8 (1.5)
Triglyceride (mg/dL), mean (SD)		147.6 (96.2)	159.6 (109.5)	141.4 (87.8)
Cholesterol (mg/dL), mean (SD)		225.1 (43.3)	223.2 (44.4)	226.0 (42.6)
High density lipoprotein (mg/dL), mean (SD)		54.1 (14.3)	51.5 (13.8)	55.4 (14.5)
Low density lipoprotein (mg/dL), mean (SD)		147.7 (39.4)	145.7 (40.1)	148.7 (39)
Taking lipid lowering medications, n (%)		960 (24.7)	328 (24.6)	632 (24.8)

**Figure 1:**
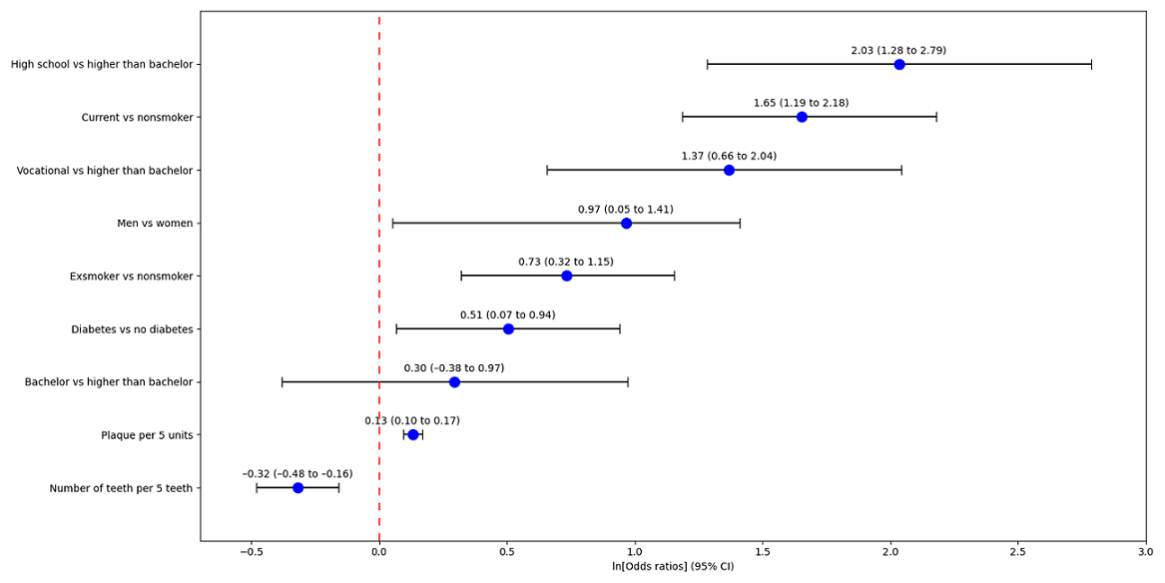
Magnitude of associations (odds ratio and 95% CI) between predictors and severe periodontitis for the mixed-effect logistic regression model.

The final multivariate MELR model included 6 features, namely, gender, education, smoking status, diabetes mellitus, number of teeth, and plaque score. The regression coefficients and odds ratios for each feature are reported in Figure 1 and Table S5 in Multimedia Appendix 1. The odds of men having severe periodontitis were 2.63 times higher than those of women. Lower levels of education were significantly associated with severe periodontitis; those educated to vocational levels and high school graduates were associated with a 3.92 and 7.59 times greater likelihood of severe periodontitis, respectively, compared to those educated above a bachelor degree. Current and exsmokers had 5.38 and 2.09 times higher odds of severe periodontitis, respectively, than nonsmokers. Participants with diabetes had a 66% greater risk of severe periodontitis compared to those without diabetes. The risk of periodontitis increased by 3% per unit increase in plaque score, in contrast to a 6% reduction in risk for every remaining tooth.

Model performance was evaluated with both the development and validation data sets (Table 3). For the development data set, the AUC (95% CI), *F*_1_-score, and Brier score for the MELR model were 0.980 (0.977-0.984), 0.869, and 0.061, respectively. The corresponding values for the validation set were 0.983 (0.977-0.989), 0.878, and 0.058, respectively, indicating that the model performed well in both data sets. The LR+ (95% CI) values (at the threshold of 0.35) were 9.4 (8.2-10.8) and 11.9 (8.8-16.3) for the development and validation data sets, respectively. This could be interpreted as participants being approximately 9-fold more likely to have periodontitis given that the model classified them as positive (ie, estimated probability ≥0.35).

The RNN model yielded AUC (95% CI) values of 0.747 (0.727-0.766) and 0.712 (0.669-0.754) for the development and validation data sets, respectively. The corresponding LR+ (95% CI) values were 2.3 (2.1-2.5) and 2.1 (1.8-2.6), respectively, which were much lower compared to those from the MELR model. The AUC (95% CI) values for the ME-SVM model were 0.761 (0.754-0.766) and 0.698 (0.681-0.734) for the development and validation data sets, respectively, with corresponding LR+ (95% CI) values of 3.1 (2.7-3.4) and 2.1 (1.7-2.6), respectively. For the ME-DT model, the AUC (95% CI) and LR+ (95% CI) values were 0.695 (0.677-0.714) and 2.4 (2.1-2.6), respectively, for development data set and 0.662 (0.621-0.702) and 2.4 (1.9-3.0), respectively, for the validation data set. The receiver operating characteristics curves for all models are shown in Figure S4 in Multimedia Appendix 1.

**Table 3:** Performances of the predictive models in the development and validation data sets.

Metric	MELR^a^			RNN^b^			ME-SVM^c^			ME-DT^d^	
	Development	Validation	Development		Validation	Development		Validation	Development		Validation
Sensitivity, % (95% CI)	91.2 (89.4-92.8)	89.4 (85-92.8)	61.6 (58.3 64.9)		54.9 (48-61.7)	52.8 (49.5-56)		46.1 (39.1-53.2)	47 (44-50.1)		44.5 (38.4-50.7)
Specificity, % (95% CI)	90.3 (88.9-91.6)	92.5 (89.9-94.7)	72.9 (70.9-75)		74.4 (70.1-78.3)	82.7 (80.9-84.4)		78.2 (74.2-81.8)	80.2 (78.4-81.9)		81.3 (77.6-84.6)
Accuracy, % (95% CI)	90.6 (89.5-91.6)	91.4 (89.2-93.3)	69.3 (67.6-71.1)		68.2 (64.5-71.7)	72.7 (71-74.4)		68.6 (65-72.1)	68.8 (67.1 – 70.4)		68.7 (65.3-72)
AUC^e^ (95% CI)	0.980 (0.977-0.984)	0.983 (0.977-0.989)	0.747 (0.727-0.766)		0.712 (0.669-0.754)	0.761 (0.754-0.766)		0.698 (0.681-0.734)	0.695 (0.677-0.714)		0.662 (0.621-0.702)
*F*_1_-score	0.869	0.878	0.573		0.543	0.564		0.467	0.509		0.493
Brier score	0.061	0.058	0.181		0.187	0.198		0.200	0.236		0.240
LR+^f^ (95% CI)	9.4 (8.2-10.8)	11.9 (8.8-16.3)	2.3 (2.1-2.5)		2.1 (1.8-2.6)	3.1 (2.7-3.4)		2.1 (1.7-2.6)	2.4 (2.1-2.6)		2.4 (1.9-3.0)

^a^MELR: mixed-effects logistic regression.

^b^RNN: recurrent neural networks.

^c^ME-SVM: mixed-effects support vector machine.

^d^ME-DT: mixed-effects decision tree.

^e^AUC: area under the receiver operating characteristic curve.

^f^LR+: positive likelihood ratio.

## Discussion

### Principal Results

Our study developed risk prediction models for periodontitis using traditional statistical and machine learning approaches. The MELR model performed best with an AUC value of 0.983 in comparison to 2 machine learning approaches, RNN and ME-SVM, which had fair performances with AUC values of 0.712 and 0.698, respectively. In addition, the Brier scores for the RNN and ME-SVM were similarly high at 0.187 to 0.200, in contrast to a score of 0.058 for the MELR, which reflects an overfitting for both machine learning models compared to the MELR. Furthermore, a LR+ value as low as 2-3 for the machine learning approaches contrasted the high value of 11.9 for the MELR.

### Comparison With Prior Work

Our MELR model also performed better than previous predictive models that applied logistic regression (AUC=0.71) [10] and was superior even to those that included salivary biomarkers, such as chitinase and protease activity (AUC=0.91) [18]. Our analyses suggest that the mixed model approach performs better than logistic regression because the former considers latent participant-specific variability and thus better captures information about population average effects of the risk features than regression approaches that use cross-sectional data.

The machine learning models (RNN, ME-SVM, and ME-DT) may have performed less well in comparison to the MELR model due to a data imbalance, as one-third of our study participants had severe periodontitis. Ling and Victor [34] suggested that a classification imbalance may affect model performance if the cost of the 2 errors (ie, false positive and false negative in the binary classification) is not the same, or if the class distribution in the validation data is different from that in the development data. The prevalence of severe periodontitis in the development and validation sets was very similar (34.4% and 34.1%, respectively; Table S6 in Multimedia Appendix 1), and this was similar to the 8th National Oral Health Survey of Thailand (2017) [25], which reported a prevalence of 26% in adults and 36% in older individuals. Thus, both data sets had similar distributions of participants with severe periodontitis that accurately reflected the overall prevalence in Thailand.

To simulate the improved performance of the MELR model, a framework to include repeated measures and random effects was applied to the machine learning models, which is a recognized advantage of the ME-ML model, although the model still failed to meet the performance levels of MELR. This may have resulted from differences in the optimization and estimation of fixed and random effects within these models; for example, the penalized quasilikelihood method was used for MELR [35] and the expectation-maximization was applied in the ME-ML [29,36]. This framework could be beneficial in estimating nonlinear relationships between predictors and outcomes; however, further studies are necessary to independently validate this.

### Strengths and Limitations

A cut off value of 0.35 was selected to reflect the observed prevalence of the condition as the uniform decision threshold and applied to all 4 screening models to enable cross-model comparisons, but this can be adjusted depending on the objective and outcome [37]. In most clinical screening or diagnostic tools, it is unlikely that the consequences of a false positive and false negative are similar. By reducing the decision threshold, participants with a lower probability would be considered positive, increasing the sensitivity (and consequently the number of false positives) but reducing the specificity (number of false negative cases). In mass screening situations, participants identified as positive would be referred for further examination; therefore, this would lead to increased numbers of participants requiring comprehensive periodontal probing. However, it would also fulfil the purpose of screening by providing early diagnosis and prompt referral by reducing the number of positive participants incorrectly identified as negative. Despite the reduced specificity associated with a lower decision threshold, this approach would identify those at greatest risk while reducing the overall workload for examiners and facilitating a more efficient allocation of resources.

While MELR models can be calculated manually as a linear combination of features, machine learning approaches must be exported in hierarchical file formats for use in websites or applications, which can be developed with a user-friendly interface. Data collection and mining from electronic care records can be combined and reformatted for more complex procedures, such as feature engineering and data preprocessing. Risk prediction modelling would therefore be amenable to data updates, facilitating model refinement, with potential portability through web- or desktop-based applications that could be provided to health care staff.

This study had several limitations. Only internal validation was carried out for model evaluation, and external validation using data from other centers or surveys is required. Furthermore, data from other Thai populations, as well as from different countries or ethnicities, would help determine the generalizability of the findings. Machine learning approaches in particular would benefit from further model refinement with larger, better characterized data sets since their performance depends on the data quality of the development sets. Although the clinical features included in the MELR model were relatively easy to examine, the assessment of a plaque score is manually intensive. Although periodontal probing is not required, the examination includes oral rinsing with plaque disclosing solutions and manual counting of the stained surfaces. Self-reporting would not be optimal since improper application and poor assessment would lead to an unreliable scoring and subsequent risk underestimation by the model. The oral features included in developing the models were limited to the factors collected by the EGAT survey. Previous studies have suggested that the inclusion of more relevant oral characteristics, such as tooth mobility and gum bleeding, would increase the performance even further, although an AUC of 0.98 is considered excellent.

### Conclusions

In conclusion, the MELR approach performed excellently, and to our knowledge represents one of the best screening models for severe periodontitis. Machine learning approaches demonstrated fair performance despite their ability to estimate nonlinear relationships. Instead of relying on PPD measurements obtained through an extensive periodontal assessment, which can be time-consuming and resource-intensive, the MELR model might be useful in health information systems to monitor oral health, prompting patients to visit a dental professional for comprehensive examination and appropriate treatment. With further independent model external validation, such a tool should be evaluated in a primary care setting to assist dental professionals in the screening of severe periodontitis to improve and direct resource allocation to where it is needed most.

## Appendix

Multimedia Appendix 1 Supplementary tables and figures.

## Abbreviations

AUC: area under the receiver operating curve
CDC-AAP: Centre for Disease Control–American Academy of Periodontology
EGAT: Electricity Generating Authority of Thailand
LR+: positive likelihood ratio
MELR: mixed-effects logistic regression
ME-DT: mixed-effects decision tree
ME-SVM: mixed-effects support vector machine
MICE: multiple imputation with chain equations
PPD: probing pocket depth
RE: gingival recession
RNN: recurrent neural networks

## Data Availability

The data sets generated and analyzed in this study are available from the corresponding author on reasonable request. Codes generated for the whole analyses are available [38].
