# Supplementary material for: Development of Risk Prediction Models for Severe Periodontitis in a Thai Population: Statistical and Machine Learning Approaches
Source: JMIR Form Res. 2023 Dec 14;7:e48351. doi: 10.2196/48351 (PMC10755655; doi:10.2196/48351)
Supplement: Multimedia Appendix 1 [file formative_v7i1e48351_app1.docx]

SUPPLEMENTARY DOCUMENT

TABLES

Table S1: Description of missing features and missing data patterns

Table S2: Description of predictors used in the multivariate imputation by chain equation of each missing features

Table S3: Comparison of distribution between imputation and complete-case data

Table S4: Univariate analysis of risk factors associated with severe periodontitis based on analysis of imputed data: A mixed-effects logistic regression

Table S5: Comparisons of estimated coefficients based on analysis using complete case and imputed data: A mixed-effects logistic regression

Table S6: Demographics and clinical characteristics for development and validation cohorts

FIGURES

Figure S1: Model convergence or iteration for each imputed feature

Figure S2: Distribution density plots of complete-case and imputed data

Figure S3: Model development framework

Figure S4: Area under receiver operating characteristics curves of the predictive models in train and test data

a) Mixed Effects Logistic Regression

b) Recurrent Neural Networks

c) Mixed Effects Support Vector Machine

d) Mixed Effects Decision Tree

MISSING DATA IMPUTATION

Percentages of missing data were quite rare, i.e., ranged from 0.03% to 9.3% as shown in Table 1. A multi-chain imputation equation method was applied to impute 8 continuous features using 2-level predictive mean matching, accounting for multilevel nature of our data. Predictors used in the MICE models were described in Table 2. A fraction of missing information was as low as 0.0005; so, five imputation data have been constructed and should be valid for further estimations. There is no exact rule to determine the number of iterations for each dataset; instead, it is visualized in Figure 1 to determine the number at which it stops trending indicating at the iteration of 8 for this data.

Distributions of complete case and imputed data are described and compared, see Table 3 and Figure 2. This suggested that all 8 features had similar means estimated based on both data sets but their SDs were little lower in imputed data than complete data. Results of analyses for coefficient estimations and tests were not much different, see Table 5.

Table S1: Description of missing features and missing data patterns

| Features | Missing from N = 3,883 | Missing Pattern |
| --- | --- | --- |
| Body mass index | 1 (0.03) | 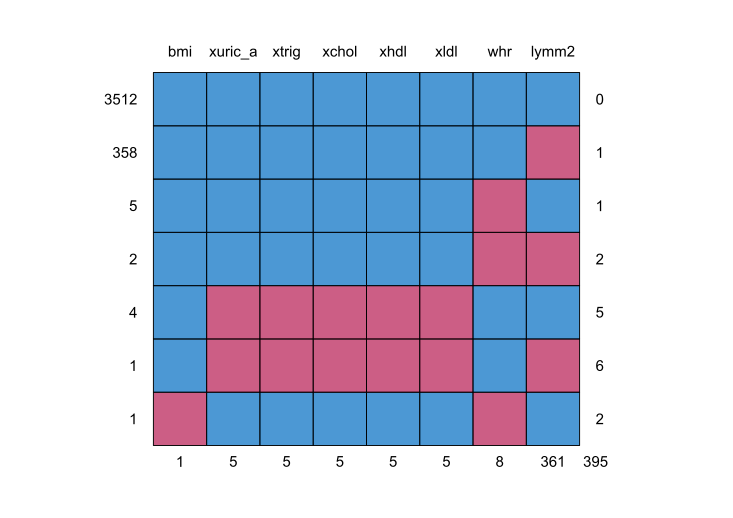 |
| Waist to hip ratio | 8 (0.20) |  |
| Lymphocytes | 361 (9.30) |  |
| Uric acid | 5 (0.13) |  |
| Triglyceride | 5 (0.13) |  |
| Cholesterol | 5 (0.13) |  |
| High density lipoprotein | 5 (0.13) |  |
| Low density lipoprotein | 5 (0.13) |  |

Abbreviations: N: number of observations.

Table S2: Description of predictors used in the multivariate imputation by chain equation of each missing features

| Features | | *Age* | | *Gender* | | *Education* | | *Income* | | *BMI* | | *WHR* | | *DM* | | *HT* | | *DLP* | | *CKD* | | *Smoking* | | *Alcohol* | | *No. of teeth* | | *Plaque S.* | | *Lymph.* | | *Uric Acid* | | *Triglyceride* | | *Cholesterol* | | *HDL* | | *LDL* | | *Lipid drugs* | | *Perio.Dx.* | |
| --- | --- | --- | --- | --- | --- | --- | --- | --- | --- | --- | --- | --- | --- | --- | --- | --- | --- | --- | --- | --- | --- | --- | --- | --- | --- | --- | --- | --- | --- | --- | --- | --- | --- | --- | --- | --- | --- | --- | --- | --- | --- | --- | --- | --- | --- |
| BMI | | ✔ | | ✔ | | ✔ | | ✔ | | ✔ | | ✔ | | ✔ | | ✔ | | ✔ | | ✔ | |  | |  | |  | |  | |  | |  | |  | |  | |  | |  | | ✔ | |  | |
| WHR | | ✔ | | ✔ | | ✔ | | ✔ | | ✔ | | ✔ | | ✔ | | ✔ | | ✔ | | ✔ | |  | |  | |  | |  | |  | |  | |  | |  | |  | |  | | ✔ | |  | |
| Lymphocytes | | ✔ | | ✔ | | ✔ | | ✔ | | ✔ | | ✔ | | ✔ | | ✔ | | ✔ | | ✔ | |  | |  | |  | |  | |  | | ✔ | | ✔ | | ✔ | | ✔ | | ✔ | | ✔ | |  | |
| Uric Acid | | ✔ | | ✔ | | ✔ | | ✔ | | ✔ | | ✔ | | ✔ | | ✔ | | ✔ | | ✔ | |  | |  | |  | |  | | ✔ | |  | | ✔ | | ✔ | |  | |  | | ✔ | |  | |
| Triglyceride | | ✔ | | ✔ | | ✔ | | ✔ | | ✔ | | ✔ | | ✔ | | ✔ | | ✔ | | ✔ | |  | |  | |  | |  | |  | |  | |  | | ✔ | | ✔ | | ✔ | | ✔ | |  | |
| Cholesterol | | ✔ | | ✔ | | ✔ | | ✔ | | ✔ | | ✔ | | ✔ | | ✔ | | ✔ | | ✔ | |  | |  | |  | |  | |  | |  | | ✔ | |  | | ✔ | | ✔ | | ✔ | |  | |
| HDL | ✔ | | ✔ | | ✔ | | ✔ | | ✔ | | ✔ | | ✔ | | ✔ | | ✔ | | ✔ | |  | |  | |  | |  | |  | |  | | ✔ | | ✔ | |  | | ✔ | | ✔ | |  | |  |
| LDL | ✔ | | ✔ | | ✔ | | ✔ | | ✔ | | ✔ | | ✔ | | ✔ | | ✔ | | ✔ | |  | |  | |  | |  | |  | |  | | ✔ | | ✔ | | ✔ | |  | | ✔ | |  | |  |

Abbreviations: BMI: Body Mass Index; CKD: Chronic Kidney Disease; DLP: Dyslipidemia; DM: Diabetes Mellitus; HDL: High Density Lipoprotein; HT: Hypertension; LDL: Low Density Lipoprotein; No. of teeth: Number of present/remaining teeth; Perio.Dx.: Severe Periodontitis; Plaque S.: Plaque Score; WHR: Waist to Hip Ratio.

Table S3: Comparison of distribution between imputation and complete-case data

| Features | Complete data distribution | Imputation distribution |
| --- | --- | --- |
| Body mass index, mean (SD) | 24.9 (3.7) | 24.9 (3.7) |
| Waist to hip ratio, mean (SD) | 0.9 (0.1) | 0.9 (0.1) |
| Lymphocytes, mean (SD), mm3 | 2158.7 (648.6) | 2156.3 (623.0) |
| Uric acid, mean (SD) | 5.9 (1.4) | 5.9 (1.4) |
| Triglyceride, mean (SD), mg/dL | 148.4 (97.7) | 147.6 (96.2) |
| Cholesterol, mean (SD), mg/dL | 225.8 (43.6) | 225.1 (43.3) |
| High density lipoprotein, mean (SD), mg/dL | 54.1 (14.3) | 54.1 (14.3) |
| Low density lipoprotein, mean (SD), mg/dL | 148.0 (39.7) | 147.7 (39.4) |

Abbreviations: mg/dL: Milligrams Per Decilitre; mm3: Per Cubic Millimetre; SD : Standard Deviation.

Table S4. Univariate analysis of risk factors associated with severe periodontitis^a^ based on analysis of imputed data: A mixed-effects logistic regression

| Features | Univariate analysis | |
| --- | --- | --- |
| Characteristics | **β (SE)** | **P-value** |
| Age | 0.06 (0.01) | 1.65 x 10^-5^ |
| Gender |  |  |
| Men | 2.05 (021) | 1.61 x 10^-23^ |
| Women | Ref |  |
| Education level |  |  |
| High school graduate or lower | 2.54 (0.38) | 7.02 x 10^-7^ |
| Vocational school graduate | 1.15 (0.35) | 3.15 x 10^-25^ |
| Bachelor’s degree graduate | 0.19 (0.35) | 8.26 x 10^-17^ |
| Above Bachelor’s degree | Ref |  |
| Monthly income, baht pm |  |  |
| Less than 20,000 | 0.31 (0.53) | 1.95 X 10^-6^ |
| Between 20,000 and 49,999 | 0.31 (0.23) | 1.08 X 10^-9^ |
| More than 50,000 | Ref |  |
| Body mass index | 0.01 (0.02) | 0.58 |
| Waist to hip ratio | 8.72 (1.12) | 5.46 x 10^-15^ |
| Diabetes mellitus | 1.22 (0.21) | 7.11 x 10^-9^ |
| Hypertension | 0.54 (0.12) | 4.52 x 10^-5^ |
| Dyslipidemia | -0.02 (0.16) | 0.39 |
| Chronic kidney disease | -0.03 (1.31) | 0.44 |
| Smoking status |  |  |
| Non-smoker | Ref |  |
| Ex-smoker | 1.56 (0.20) | 5.08 x 10^-15^ |
| Current smoker | 3.14 (0.27) | 2.23 x 10-^31^ |
| Alcohol consumption |  |  |
| Non-consumer | Ref |  |
| Ex-consumer | 0.89 (0.20) | 6.63 x 10^-6^ |
| Current consumer | 1.31 (0.17) | 9.25 x 10^-15^ |
| Number of present/remaining teeth | -0.15 (0.02) | 4.47 x 10^-22^ |
| Plaque score | 0.04 (0.003) | 6.93 x 10^-37^ |
| Lymphocytes | 0.001 (0.0001) | 4.78 x 10^-7^ |
| Uric acid | 0.22 (0.05) | 1.18 x 10^-5^ |
| Triglyceride | 0.004 (0.001) | 6.56 x 10^-7^ |
| Cholesterol | - 0.001 (0.001) | 0.53 |
| High density lipoprotein | -0.04 (0.005) | 1.89 x 10^-12^ |
| Low density lipoprotein | - 0.002 (0.002) | 0.23 |
| Taking lipid lowering medications | -0.13 (0.15) | 0.40 |

a : non-severe periodontitis includes none, mild and moderate periodontitis, classified according to Centre for Disease Control – American Academy of Periodontology criteria.

Abbreviations: SE: Standard Error; Ref: Reference covariate group; β: Regression Coefficient.

Table S5: Comparisons of estimated coefficients based on analysis using complete case and imputed data: A mixed-effects logistic regression

| Features | Analysis on Complete Case Data | | Analysis on Imputed Data | |
| --- | --- | --- | --- | --- |
|  | **β (SE)** | **Odds ratio** | **β (SE)** | **Odds ratio** |
| Gender |  |  |  |  |
| Men | 0.97 (0.23) | 2.63 (1.68 to 4.10) | 0.97 (0.23) | 2.63 (1.68 to 3.81) |
| Women | Ref | Ref | Ref | Ref |
| Education level |  |  |  |  |
| High school graduate or lower | 2.04 (0.38) | 7.68 (3.62 to 16.30) | 2.03 (0.39) | 7.59 (3.56 to 13.79) |
| Vocational school graduate | 1.35 (0.35) | 3.86 (1.93 to 7.72) | 1.37 (0.36) | 3.92 (1.94 to 6.78) |
| Bachelor’s degree graduate | 0.29 (0.35) | 1.34 (0.68 to 2.64) | 0.30 (0.34) | 1.35 (0.66 to 2.61) |
| Above Bachelor’s degree | Ref | Ref | Ref | Ref |
| Smoking status |  |  |  |  |
| Current smoker | 1.68 (0.25) | 5.38 (3.28 to 8.83) | 1.65 (0.25) | 5.38 (3.64 to 9.06) |
| Ex-smoker | 0.73 (0.21) | 2.09 (1.38 to 3.17) | 0.73 (0.21) | 2.09 (1.33 to 2.82) |
| Non-smoker | Ref | Ref | Ref | Ref |
| Diabetes Mellitus | 0.50 (0.22) | 1.66 (1.07 to 2.57) | 0.51 (0.23) | 1.66 (1.11 to 2.48) |
| Number of present/remaining teeth | -0.06 (0.02) | 0.94 (0.91 to 0.97) | -0.06 (0.02) | 0.94 (0.91 to 0.96) |
| Plaque Score | 0.03 (0.004) | 1.026 (1.019 to 1.034) | 0.03 (0.004) | 1.026 (1.019 to 1.033) |
| Intercept | -3.93 (0.64) |  | -2.26 (0.67) |  |
| Variance | **5.65 (0.86)** |  | **5.64 (0.85)** |  |

Abbreviations: CI: Confidence Interval; SE: Standard Error; Ref: Reference covariate group; β: Regression Coefficient.

Table S6: Demographics and clinical characteristics for development and validation cohorts

|  | Total number of subjects/observations  (n=2,086, N=3,883) | Development  (n=1,759, N=3,112) | Validation  (n=327, N=771) |
| --- | --- | --- | --- |
| Characteristics |  |  |  |
| Age, mean (SD), years | 54.4 (5.0) | 54.4 (5.1) | 54.3 (4.9) |
| Gender, n(%) |  |  |  |
| Men | 1,482 (71.0) | 1,244 (70.7) | 238 (72.8) |
| Women | 604 (29.0) | 515 (29.3) | 89 (27.2) |
| Education level, N(%) |  |  |  |
| High school graduate or lower | 767 (19.8) | 622 (20.0) | 145 (18.8) |
| Vocational school graduate | 1,282 (33.0) | 1,025 (32.9) | 257 (33.3) |
| Bachelor’s degree graduate | 1,519 (39.1) | 1,215 (39.1) | 304 (39.4) |
| Above Bachelor’s degree | 315 (8.1) | 250 (8.0) | 65 (8.5) |
| Monthly income, N(%), baht pm |  |  |  |
| Less than 20,000 | 306 (7.9) | 258 (8.3) | 48 (6.2) |
| Between 20000 and 49,999 | 828 (21.3) | 656 (21.1) | 172 (22.3) |
| More than 50,000 | 2,749 (70.8) | 2,198 (70.6) | 551 (71.5) |
| Body mass index, mean (SD) | 24.9 (3.7) | 24.8 (3.7) | 25.0 (3.7) |
| Waist to hip ratio, mean (SD) | 0.9 (0.1) | 0.9 (0.1) | 0.9 (0.1) |
| Diabetes mellitus, N(%) | 499 (12.9) | 397 (12.8) | 102 (13.2) |
| Hypertension, N(%) | 1,741 (44.8) | 1,381 (44.4) | 360 (46.7) |
| Dyslipidemia, N(%) | 2,775 (71.5) | 2,214 (71.1) | 561 (72.8) |
| Chronic kidney disease, N(%) | 293 (7.5) | 243 (7.8) | 50 (6.5) |
| Smoking status, N(%) |  |  |  |
| Non-smoker | 2,092 (53.9) | 1,677 (53.9) | 415 (53.8) |
| Ex-smoker | 1,143 (29.4) | 906 (29.1) | 237 (30.8) |
| Current smoker | 648 (16.7) | 529 (17.0) | 119 (15.4) |
| Alcohol consumption, N(%) |  |  |  |
| Non-consumer | 1,249 (32.2) | 1,001 (32.2) | 248 (32.2) |
| Occasional consumer | 695 (17.9) | 550 (17.6) | 145 (18.8) |
| Frequent consumer | 1,939 (49.9) | 1,561 (50.2) | 378 (49.0) |
| Number of present/remaining teeth, mean (SD) | 23.4 (4.9) | 23.4 (5.0) | 23.5 (4.8) |
| Plaque score, mean (SD), percentage | 70.9 (21.5) | 71.0 (21.3) | 70.4 (22.2) |
| Lymphocytes, mean (SD), mm3 | 2,156.3 (623.0) | 2,157 (610.8) | 2,149.6 (670.5) |
| Uric acid, mean (SD) | 5.9 (1.4) | 5.9 (1.5) | 6.0 (1.4) |
| Triglyceride, mean (SD), mg/dL | 147.6 (96.2) | 147.4 (95.9) | 148.3 (97.4) |
| Cholesterol, mean (SD), mg/dL | 225.1 (43.3) | 224.8 (43.6) | 226.0 (41.9) |
| High density lipoprotein, mean (SD) , mg/dL | 54.1 (14.3) | 54.1 (14.2) | 54.0 (14.9) |
| Low density lipoprotein, mean (SD) , mg/dL | 147.7 (39.4) | 147.5 (39.7) | 148.4 (38.2) |
| Taking lipid lowering medications, N(%) | 960 (24.7) | 755 (24.3) | 205 (26.6) |
| Non-severe periodontitis ^a^, N(%) | 2,550 (65.7) | 2,042 (65.6) | 508 (65.9) |
| Severe periodontitis, N(%) | 1,333 (34.3) | 1,070 (34.4) | 263 (34.1) |

Abbreviations: mg/dL : Milligrams Per Decilitre; mm3 : Per Cubic Millimetre; n: number of subjects; N: number of observations; pm : Per Month; SD : Standard Deviation.

Figure S1: Model convergence or iteration for each imputed feature


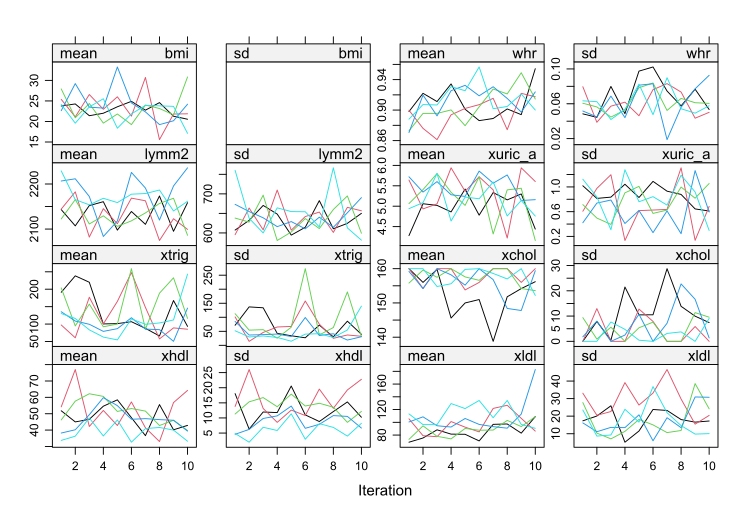


Legends: bmi: body mass index; lymm2: lymphocytes; sd: standard deviation; whr: waist-to-hip ratio; xchol: cholesterol; xhdl: high density lipoprotein; xldl: low density lipoprotein; xtrig; triglyceride; xuric_a: uric acid.

Figure S2: Distribution density plots of complete-case and imputed data


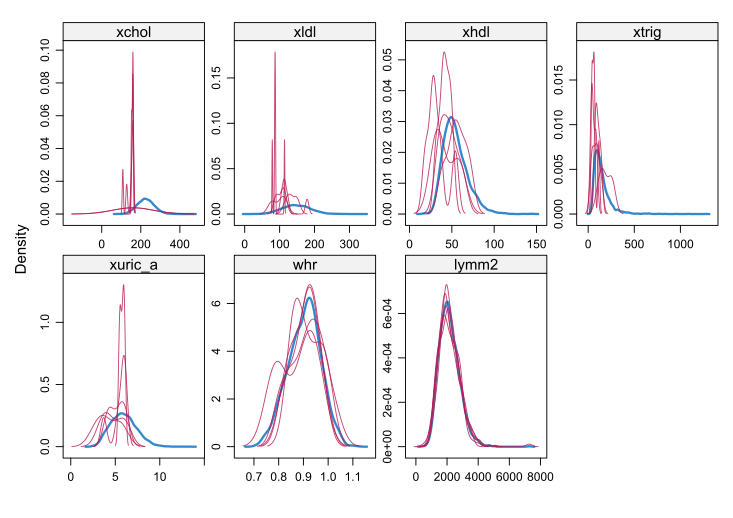


Legends: lymm2: lymphocytes; whr: waist-to-hip ratio; xchol: cholesterol; xhdl: high density lipoprotein; xldl: low density lipoprotein; xtrig; triglyceride; xuric_a: uric acid.

Figure S3: Model development framework


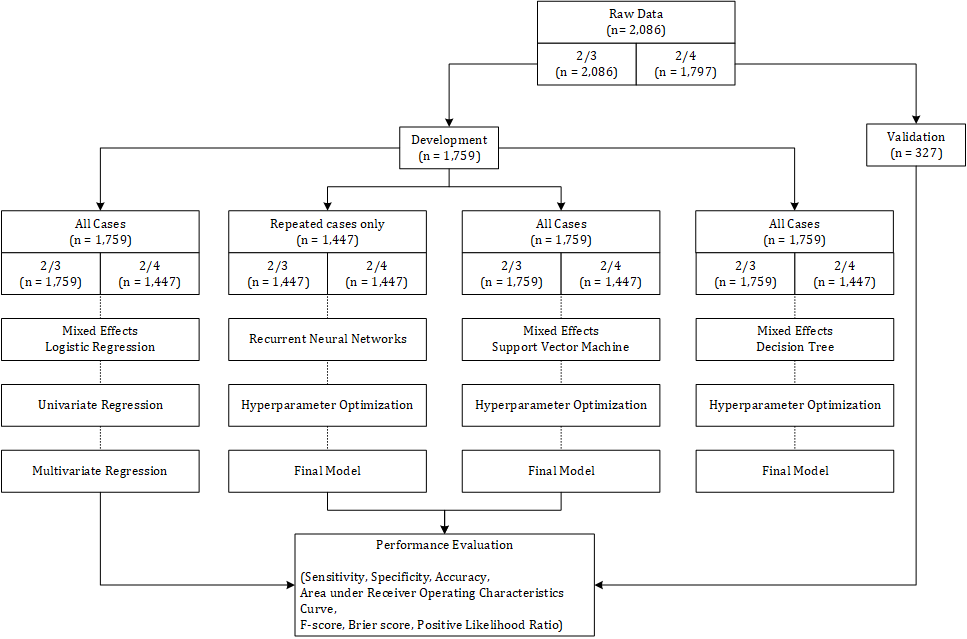


Legends: n: number of subjects.

Figure S4 : Area under receiver operating characteristics curves of the predictive models in train and test data

| 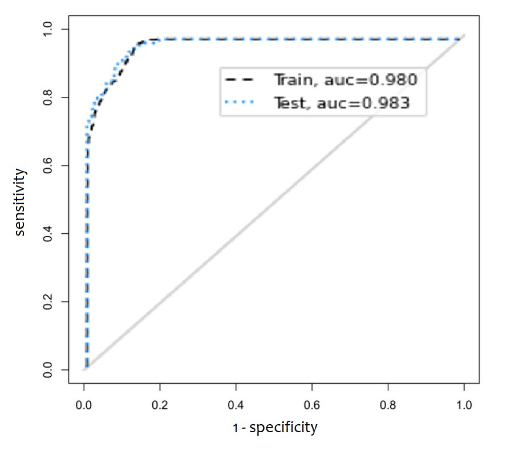  (a) Mixed Effects Logistic Regression | 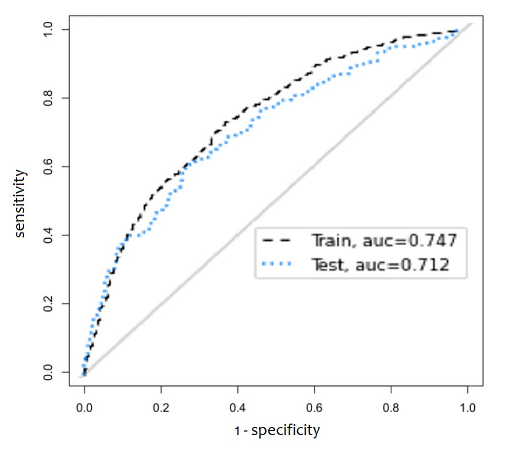  (b) Recurrent Neural Networks |
| --- | --- |
| 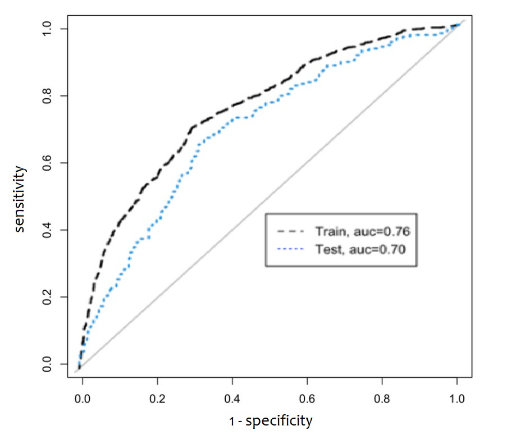  (c) Mixed Effects Support Vector Machine | 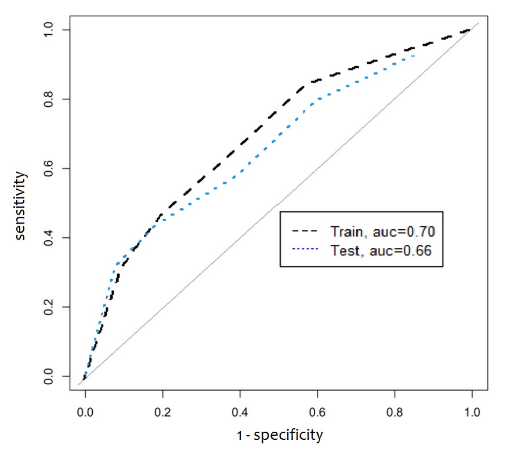  (d) Mixed Effects Decision Tree |

Legends: train: Development Dataset; test: Validation Dataset
